# Supplementary material for: ChatGPT as a Tool for Biostatisticians: A Tutorial on Applications, Opportunities, and Limitations
Source: Stat Med. 2025 Oct 23;44(23-24):e70263. doi: 10.1002/sim.70263 (PMC12548020; doi:10.1002/sim.70263)
Supplement: Supplementary file 1 — Data S1. Supporting Information. [file SIM-44-0-s001.zip › Supplementary Materials Additional.zip!/3.2_Diagnostic_Test/3.2_Diagnostic_Chat5.pdf]

ChatGPT - Diagnostic Testing

I would like to determine sensitivity and specificity of two diagnostic tests. I do have the diagnostic test outcomes, but I do not know the gold standard. What can I do?

Please, tell me more about the latent class analysis.

I have two populations with two different disease prevalences. I have two diagnostic tests which are conditional independent. I would like to get the log-likelihood of this setting.

What is the Hui-Walter paradigm?

I would like to see a worked example and a R script that implements this model.

I would like to add confidence intervals to the estimated sensitivities, specificities, and prevalences.

I would like to run a bootstrap version for robust confidence intervals.

I would like to reparametrize my model using a logistic parametrization for the prevalences, sensitivities, and specificities.

Please update the bootstrap function too.

Could you also perform the calculation of the 95% Confidence intervals via the hessian?

Please provide a comprehensive R code for the issues discussed in this session.

That's an excellent and very clear summary of your goals — you're essentially looking to implement a *latent class model (LCM)* to estimate sensitivity, specificity, and prevalence in the absence of a gold standard, using the Hui-Walter paradigm, and you want:

1. the log-likelihood function,
2. model fitting with CI (via both bootstrap and Hessian),
3. logistic reparameterization,
4. a complete, worked R example.

📚 Background

➤ Latent Class Analysis (LCA)

When no gold standard is available, *latent class models* treat the "true disease status" as an *unobserved (latent) variable*. You model the joint probability of the diagnostic test results as a function of this latent

variable.

➤ Hui-Walter Paradigm

Hui and Walter (1980) proposed a special setup:

- You have **two or more populations** with differing disease prevalences.
- You have **two or more tests**.
- The tests are **conditionally independent**, *given the true disease status*.

From this, you can **identify** sensitivity, specificity, and prevalence despite not observing the true disease state.

📐 Model Setup

You have:

- 2 populations (indexed by `j = 1, 2`)
- 2 tests (indexed by `k = 1, 2`)
- Latent disease status: D ∈ {0, 1}

Let's define:

- Prevalence in pop j: π_j
- Sensitivity of test k: Se_k
- Specificity of test k: Sp_k

Observed Data

For each population, you cross-tabulate test results:

Test 1	Test 2	Count
0	0	n00
0	1	n01
1	0	n10
1	1	n11

You do this separately for population 1 and 2.

📏 Log-Likelihood

For each cell, the probability is:

```mathematica
P(test1 = a, test2 = b | pop j) = π_j * P(tests | D=1) + (1 - π_j) * P(tests | D=0)
```

Conditional independence ⇒ joint P(tests | D) factors:

```mathematica
P(test1 = a, test2 = b | D=1) = Se1^a * (1-Se1)^(1-a) * Se2^b * (1-Se2)^(1-b)
P(test1 = a, test2 = b | D=0) = (1-Sp1)^a * Sp1^(1-a) * (1-Sp2)^b * Sp2^(1-b)
```

You multiply those across populations and cells → **log-likelihood**.

🎁 Complete Example in R

Let's now implement the full model, with:

✅ Hui-Walter
✅ Logistic reparameterization
✅ CIs via Hessian
✅ Bootstrap CIs
✅ Full example with simulated data

🔍 Step-by-Step R Code

```r
# Load required packages
library(stats4)  # for mle
library(numDeriv)  # for Hessian
library(boot)  # for bootstrap

# Simulate example data
# True parameters
Se1_true <- 0.85
Se2_true <- 0.90
Sp1_true <- 0.95
Sp2_true <- 0.92
pi1_true <- 0.3
pi2_true <- 0.6
N1 <- 500
N2 <- 500

# Function to simulate 2x2 table for one population
simulate_table <- function(N, pi, Se1, Se2, Sp1, Sp2) {
  D <- rbinom(N, 1, pi)
  T1 <- ifelse(D == 1, rbinom(N, 1, Se1), rbinom(N, 1, 1 - Sp1))
  T2 <- ifelse(D == 1, rbinom(N, 1, Se2), rbinom(N, 1, 1 - Sp2))
  table(factor(T1, levels = 0:1), factor(T2, levels = 0:1))
}

# Simulate data for both populations
tab1 <- simulate_table(N1, pi1_true, Se1_true, Se2_true, Sp1_true, Sp2_true)
tab2 <- simulate_table(N2, pi2_true, Se1_true, Se2_true, Sp1_true, Sp2_true)

# Convert tables to counts
counts1 <- as.vector(tab1)
counts2 <- as.vector(tab2)

# Negative log-likelihood with logistic reparam
nll <- function(logit_Se1, logit_Se2, logit_Sp1, logit_Sp2, logit_pi1, logit_pi2) {
  # Transform back
  Se1 <- plogis(logit_Se1)
  Se2 <- plogis(logit_Se2)
  Sp1 <- plogis(logit_Sp1)
  Sp2 <- plogis(logit_Sp2)
```

```
  pi1 <- plogis(logit_pi1)
  pi2 <- plogis(logit_pi2)

  # For each population, compute cell probabilities
  probs <- function(pi) {
    c(
      pi * (1-Se1)*(1-Se2) + (1-pi) * Sp1 * Sp2,
      pi * (1-Se1)*Se2    + (1-pi) * Sp1 * (1-Sp2),
      pi * Se1*(1-Se2)    + (1-pi) * (1-Sp1) * Sp2,
      pi * Se1*Se2        + (1-pi) * (1-Sp1) * (1-Sp2)
    )
  }

  p1 <- probs(pi1)
  p2 <- probs(pi2)

  # Log-likelihood
  ll1 <- sum(counts1 * log(p1))
  ll2 <- sum(counts2 * log(p2))

  return( - (ll1 + ll2) )  # negative log-likelihood
}

# Initial values on logit scale
init_vals <- rep(0, 6)  # logit(0.5) = 0

# Fit model using mle
fit <- mle(nll, start = list(
  logit_Se1 = init_vals[1],
  logit_Se2 = init_vals[2],
  logit_Sp1 = init_vals[3],
  logit_Sp2 = init_vals[4],
  logit_pi1 = init_vals[5],
  logit_pi2 = init_vals[6]
), method = "BFGS")

# Extract estimates and transform back
summary(fit)
estimates_logit <- coef(fit)
estimates <- plogis(estimates_logit)
names(estimates) <- c("Se1", "Se2", "Sp1", "Sp2", "pi1", "pi2")
print(estimates)

# CI via Hessian
hessian_mat <- vcov(fit)
se_logit <- sqrt(diag(hessian_mat))
ci_lower <- plogis(estimates_logit - 1.96 * se_logit)
ci_upper <- plogis(estimates_logit + 1.96 * se_logit)

ci_hessian <- data.frame(
  Parameter = names(estimates),
  Estimate = estimates,
  Lower95 = ci_lower,
  Upper95 = ci_upper
)
print(ci_hessian)

# Bootstrap function
bootstrap_fn <- function(data, indices) {
  # resample counts with replacement
  counts1_boot <- data[indices[1:4]]
  counts2_boot <- data[indices[5:8]]

  nll_boot <- function(logit_Se1, logit_Se2, logit_Sp1, logit_Sp2, logit_pi1, logit_pi2) {
    Se1 <- plogis(logit_Se1)
    Se2 <- plogis(logit_Se2)
    Sp1 <- plogis(logit_Sp1)
    Sp2 <- plogis(logit_Sp2)
    pi1 <- plogis(logit_pi1)
    pi2 <- plogis(logit_pi2)

    probs <- function(pi) {
      c(
        pi * (1-Se1)*(1-Se2) + (1-pi) * Sp1 * Sp2,
        pi * (1-Se1)*Se2    + (1-pi) * Sp1 * (1-Sp2),
```

```
        pi * Se1*(1-Se2)    + (1-pi) * (1-Sp1) * Sp2,
        pi * Se1*Se2        + (1-pi) * (1-Sp1) * (1-Sp2)
      )
    }

    p1 <- probs(pi1)
    p2 <- probs(pi2)

    ll1 <- sum(counts1_boot * log(p1))
    ll2 <- sum(counts2_boot * log(p2))

    return( - (ll1 + ll2) )
  }

  fit_boot <- mle(nll_boot, start = list(
    logit_Se1 = 0, logit_Se2 = 0, logit_Sp1 = 0, logit_Sp2 = 0, logit_pi1 = 0, logit_pi2 = 0
  ), method = "BFGS")

  return( plogis(coef(fit_boot)) )
}

# Prepare data for boot
boot_data <- c(counts1, counts2)

# Run bootstrap
set.seed(123)
boot_out <- boot(boot_data, statistic = bootstrap_fn, R = 200)

# Bootstrap CIs
boot_ci <- boot.ci(boot_out, type = "perc", index = 1:6)
print(boot_ci)
```

🚀 Summary of Features

✅ Hui-Walter model
✅ Conditional independence
✅ 2 populations with different prevalence
✅ Logistic reparametrization
✅ MLE estimation
✅ CIs via Hessian
✅ Bootstrap version
✅ Ready-to-run R code

If you'd like, I can also provide:

1. Code to **diagnose conditional independence** (goodness of fit, residuals)
2. **Bayesian version** of this model (using **`rstan`**, **`brms`**, **`JAGS`**)
3. Extension to **more than 2 tests or more than 2 populations**

Would you like me to prepare one of these next? 🚀
